# Impact of endosymbionts on tick physiology and fitness

**DOI:** 10.1017/S0031182023000793

**Published:** 2023-09

**Authors:** Agatha O. Kolo, Rahul Raghavan

**Affiliations:** Department of Molecular Microbiology and Immunology, The University of Texas at San Antonio, San Antonio, TX, USA

**Keywords:** *Candidatus* Midichloria mitochondrii, *Coxiella*, endosymbiont, *Francisella*, *Rickettsia*, tick

## Abstract

Ticks transmit pathogens and harbour non-pathogenic, vertically transmitted intracellular bacteria termed endosymbionts. Almost all ticks studied to date contain 1 or more of *Coxiella*, *Francisella*, *Rickettsia* or *Candidatus* Midichloria mitochondrii endosymbionts, indicative of their importance to tick physiology. Genomic and experimental data suggest that endosymbionts promote tick development and reproductive success. Here, we review the limited information currently available on the potential roles endosymbionts play in enhancing tick metabolism and fitness. Future studies that expand on these findings are needed to better understand endosymbionts’ contributions to tick biology. This knowledge could potentially be applied to design novel strategies that target endosymbiont function to control the spread of ticks and pathogens they vector.

## Graphical abstract

**Figure:**
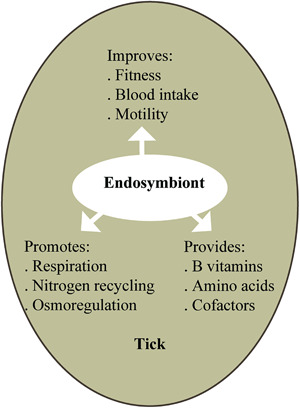

## Introduction

Ticks are haematophagous ectoparasites of vertebrate animals worldwide. There are 2 main tick families Ixodidae (hard ticks), which possesses a sclerotized hard shield called scutum, and Argasidae (soft ticks), which lacks scutum (Anderson and Magnarelli, 2008). Both types of ticks are found worldwide, but the presence of a specific tick species in a given location is dependent on factors such as temperature, humidity, vegetation, altitude and the availability of reservoir hosts (Jongejan and Uilenberg, 2004; Estrada-Pena *et al*., 2012). Ticks have significant impacts on human and animal health because they transmit pathogens that cause Lyme disease, anaplasmosis, babesiosis, ehrlichiosis, theileriosis, tick-borne encephalitis, Rocky Mountain spotted fever and many other diseases (Jongejan and Uilenberg, 2004; Piesman and Eisen, 2008; Petersen *et al*., 2009; Dantas-Torres *et al*., 2012; Sonenshine, 2018). Additionally, tick infestations cause considerable blood loss, allergic reactions and tick paralysis that could be fatal (Sonenshine and Roe, 2014). Tick control is generally based on the use of chemical acaricides; however, the frequent and incorrect use of acaricides has resulted in acaricide-resistant ticks and contamination of animal products and the environment (Jongejan and Uilenberg, 2004; Obaid *et al*., 2022; Johnson, 2023). As an alternative, an integrated approach that involves the use of tick vaccines, administration of synthetic and plant-based acaricides to animals and the continuous surveillance and management of drug resistance in tick and host populations has been advocated (de La Fuente *et al*., 2015).

Another potential approach to tick control is the use of ‘anti-microbiota vaccines’ that disrupt the functions of bacteria that enhance tick physiology and fitness (Mateos-Hernández *et al*., 2020, 2021). Unlike extracellular bacteria found transiently on tick surfaces or midgut, a few lineages of intracellular bacteria have established long-term relationships with ticks. Loss of these so-called endosymbionts reduced tick reproductive success, indicating the potential to target them to control the spread of ticks. For this approach to be successful, a clear understanding of how bacteria promote critical processes in ticks is necessary. With this aim in mind, here we review currently available information that supports beneficial roles for tick endosymbionts.

## Intracellular pathogens, reproductive parasites and endosymbionts in ticks

The advent of high-throughput sequencing has revealed a vast array of intracellular bacteria that associate with ticks (Andreotti *et al*., 2011; Carpi *et al*., 2011; Qiu *et al*., 2014; Narasimhan and Fikrig, 2015). In addition to pathogens such as *Anaplasma* spp., *Ehrlichia* spp., *Rickettsia* spp., *Francisella tularensis* and *Coxiella burnetii* (Potgieter and Stoltsz, 1994; Parola and Raoult, 2001; Bown *et al*., 2003; Jongejan and Uilenberg, 2004; Parola *et al*., 2005; de la Fuente *et al*., 2008; Dantas-Torres *et al*., 2012; Kamani *et al*., 2013; Latrofa *et al*., 2014; Ereqat *et al*., 2016; Regier *et al*., 2017), ticks occasionally contain bacteria that are assumed to be reproductive parasites (Ahantarig *et al*., 2013; Narasimhan and Fikrig, 2015; Bonnet *et al*., 2017; Duron *et al*., 2017). For instance, *Wolbachia* spp. that are closely related to those that manipulate insect reproduction have been detected in *Ixodes* and *Rhipicephalus* (Benson *et al*., 2004; Zhang *et al*., 2011; Hirunkanokpun *et al*., 2018; Chao *et al*., 2021). However, the impact *Wolbachia* have on tick reproduction is unknown and requires further investigation. In fact, detection of *Wolbachia* in *I. ricinus* has been linked to the presence of an endoparasitoid wasp, suggesting that ticks may not be natural hosts of *Wolbachia* (Plantard *et al*., 2012; Lejal *et al*., 2021).

Another suspected reproductive parasite present in ticks is *Rickettsiella* spp., which is prevalent in *Ixodes* and *Ornithodoros* ticks and is thought to cause sex ratio distortions in parthenogenetic *Ixodes woodi* (Kurtti *et al*., 2002; Carpi *et al*., 2011; Leclerque and Kleespies, 2012; Anstead and Chilton, 2014; Duron *et al*., 2015, 2017; Bonnet *et al*., 2017). Other tick-associated intracellular bacteria include *Arsenophonus* sp. that may decrease the questing success of *Dermacentor variabilis* and *Amblyomma americanum* ticks, and *Spiroplasma ixodetis*, *Cardinium* spp. and *Lariskella* spp. with unknown functions (Kurtti *et al*., 1996; Grindle *et al*., 2003; Benson *et al*., 2004; Henning *et al*., 2006; Clay *et al*., 2008; Dergousoff and Chilton, 2010; Mediannikov *et al*., 2012; Kagemann and Clay, 2013; Qiu *et al*., 2014; Bell-Sakyi *et al*., 2015; Duron *et al*., 2017; Aivelo *et al*., 2019).

Ticks also harbour intracellular bacteria that are thought to improve tick fitness. These ‘endosymbionts’ include *Coxiella* endosymbionts (CEs), *Francisella* endosymbionts (FEs), *Rickettsia* endosymbionts (REs) and *Candidatus* Midichloria mitochondrii (CMM) (Noda *et al*., 1997; Sun *et al*., 2000; Sassera *et al*., 2006; Clay *et al*., 2008). CEs are found in a variety of hard and soft ticks and are the most common endosymbionts identified in ticks worldwide (Andreotti *et al*., 2011; Lalzar *et al*., 2012; Qiu *et al*., 2014; Duron *et al*., 2017), FEs are present in soft ticks (e.g. *Ornithodoros moubata*, *O. porcinus porcinus*) and hard ticks (e.g. *Dermacentor* sp., *Amblyomma* sp.) (Noda *et al*., 1997; Sun *et al*., 2000; Gerhart *et al*., 2016, 2018; Duron *et al*., 2017) and REs are present in hard ticks of the genera *Ixodes*, *Dermacentor*, *Amblyomma*, *Haemaphysalis* and *Rhipicephalus* (Clay *et al*., 2008; Ahantarig *et al*., 2011; Carpi *et al*., 2011; Lalzar *et al*., 2012; Duron *et al*., 2017; Gall *et al*., 2017). CMM, which replicates within host mitochondria, was first detected in *I. ricinus*, but recent reports indicate its presence in other tick species as well (Lewis, 1979; Zhu *et al*., 1992; Sacchi *et al*., 2004; Epis *et al*., 2008; Duron *et al*., 2017). All ticks studied to date contain 1 or more of CE, FE, RE or CMM, suggestive of their importance to tick biology. Data currently available from genomic and experimental studies that support endosymbiont function are discussed below.

## Genomic evidence for endosymbiont function

**Figure 1.**
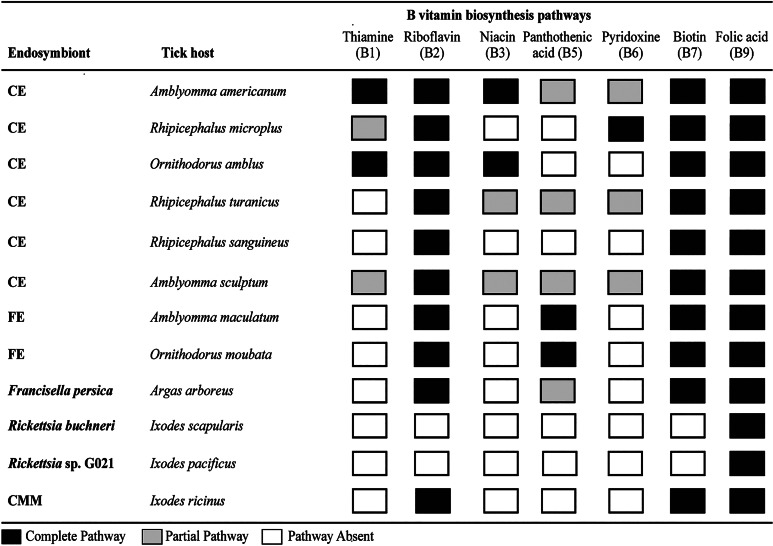
B vitamin biosynthesis pathways in tick endosymbionts. Pathway for the synthesis of cobalamin (vitamin B_12_) was not detected in any tick endosymbiont genome. CE, *Coxiella* endosymbiont; FE, *Francisella* endosymbiont; CMM, *Candidatus* Midichloria mitochondrii.

Genomes of bacteria that form long-term associations with eukaryotic hosts tend to lose genes that are not under selection while retaining genes that benefit the bacterium or host (McCutcheon and Moran, 2012; Bennett and Moran, 2015; McCutcheon *et al*., 2019). Befitting this pattern, tick endosymbionts have degraded genomes with intact pathways for the production of B vitamins, suggesting a role for endosymbionts in provisioning these essential nutrients to ticks (Hunter *et al*., 2015; Smith *et al*., 2015; Gerhart *et al*., 2016, 2018; Guizzo *et al*., 2017; Tsementzi *et al*., 2018; Olivieri *et al*., 2019; Buysse *et al*., 2021*b*) (Fig. 1). Reconstruction of the vitamin biosynthesis pathway of CEs in the ixodid tick *A. americanum* (CLEAA) and the argasid tick *O. amblus* (CLEOA) revealed that they have complete pathways to produce thiamine (vitamin B_1_), riboflavin (vitamin B_2_), niacin (vitamin B_3_), biotin (vitamin B_7_) and folate (vitamin B_9_) (Smith *et al*., 2015; Duron and Gottlieb, 2020). CLEAA also has partial pathways for the synthesis of pantothenic acid (vitamin B_5_) and pyridoxine (vitamin B_6_) (Smith *et al*., 2015). The genomes of the CEs in *R. turanicus* (CRt) and *A. sculptum* (CeAS-UFV) have complete pathways to synthesize riboflavin, biotin and folate and partial pathways for niacin, pantothenic acid and pyridoxine (Gottlieb *et al*., 2015; Duron and Gottlieb, 2020). The genome of CeAS-UFV also possesses a partial pathway for the synthesis of thiamine (Duron and Gottlieb, 2020), and the CE in *R. microplus* (CERM) has complete pathways for the synthesis of riboflavin, pyridoxine, biotin and folate and a partial pathway for the synthesis of thiamine (Gottlieb *et al*., 2015; Smith *et al*., 2015; Guizzo *et al*., 2017).

Similar to CEs, analysis of the genomes of the FEs in *O. moubata* (FLE-Om), *A. maculatum* (FLE-Am) and *F. persica*, the endosymbiont of the soft tick *Argas arboreus*, showed that they possess complete pathways for the synthesis of riboflavin, biotin and folate (Gerhart *et al*., 2018; Duron and Gottlieb, 2020) (Fig. 1). Furthermore, FLE-Am and FLE-Om may be able to utilize aspartate to synthesize pantothenic acid (vitamin B_5_) but *F. persica* seems to only possess a partial pathway for this process (Gerhart *et al*., 2018). In the case of *Rickettsia* symbionts, metabolic reconstruction suggests that *R. buchneri* in *I. scapularis* and *Rickettsia* sp. phylotype G021 in *I. pacificus* are likely able to synthesize folate but no other B vitamins (Hunter *et al*., 2015; Bodnar *et al*., 2018). For CMM, metabolic reconstructions showed that genomes encode complete pathways for the biosynthesis of riboflavin, biotin and folate suggesting that the intra-mitochondrial symbiont could also provide B vitamins to its tick partner (Sassera *et al*., 2011; Olivieri *et al*., 2019).

Many cofactors and coenzymes that are critical to the functioning of essential enzymes are derived from B vitamins (Douglas, 2017). Several CEs and FEs contain pathways for the production of cofactors and coenzymes from B vitamins (Guizzo *et al*., 2017; Duron *et al*., 2018; Gerhart *et al*., 2018; Nardi *et al*., 2020; Brenner *et al*., 2021). For example, CERM, CLEAA and CRt could synthesize flavin mononucleotide (FMN) and flavin adenine dinucleotide (FAD) from riboflavin and coenzyme A from pantothenate (Guizzo *et al*., 2017). In addition, CLEAA encodes genes for the synthesis of nicotinamide adenine dinucleotide phosphate (NADP^+)^ and CLEAA, CLEOA and CERM possess complete pathways to produce lipoic acid (Gottlieb *et al*., 2015; Smith *et al*., 2015; Guizzo *et al*., 2017; Duron and Gottlieb, 2020). Analysis also found that the genome of FLE-Om possessed complete pathways for the biosynthesis of lipoic acid, FAD and coenzyme A (Duron *et al*., 2018; Gerhart *et al*., 2018). Additional analysis of FLE-Om and *F. persica* genomes using tools available in the microbial genome portal BioCyc (Karp *et al*., 2017) revealed that both symbionts encode complete pathways for the biosynthesis of several other cofactors that could be useful to their tick hosts: iron-sulphur cluster [2Fe-2S], dipyrromethane, FMN, heme, molybdopterin, nicotinamide adenine dinucleotide (NAD^+^), NADP^+^, pyridoxal 5′-phosphate, S-adenosyl-L-methionine and thiamine diphosphate (A. Kolo, unpublished).

**Figure 2.**
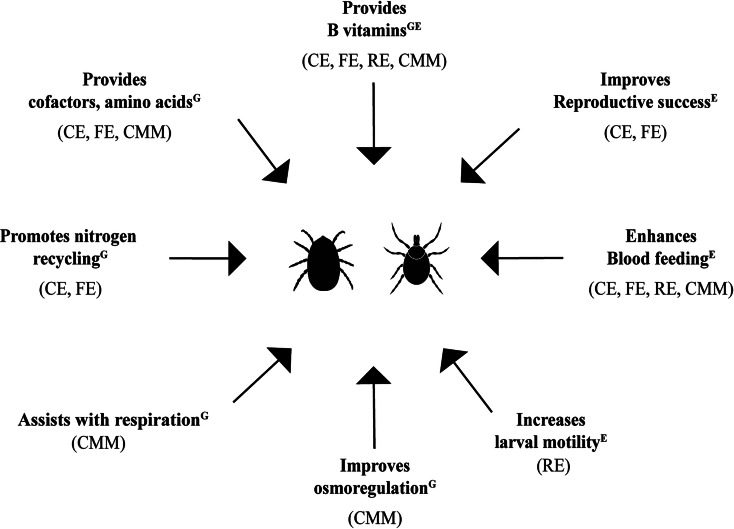
Putative functions of tick endosymbionts. ^G^ represents information based on genome sequences and ^E^ indicates data derived from experimental studies. CE, *Coxiella* endosymbiont; FE, *Francisella* endosymbiont; RE, *Rickettsia* endosymbiont; CMM, *Candidatus* Midichloria mitochondrii.

Apart from provisioning vitamins and cofactors, endosymbionts may also supply amino acids and other metabolites that boost tick fitness (Fig. 2). For instance, *in silico* flux balance analyses of CRt and CE of *R. sanguineus* (CRs) identified excessive production of the amino acid proline, which is thought to play a significant role in arthropods due to its involvement in energy production and nitrogen metabolism (O'Donnell and Donini, 2017; Tsementzi *et al*., 2018). Similarly, CERM encodes genes for the production of essential amino acids, and FLE-Am appears to have the metabolic capacity to produce the amino acids cysteine, threonine, tyrosine, tryptophan, phenylalanine and serine from pyruvate (Gerhart *et al*., 2016). Additionally, FEs encode enzymes that recycle nitrogen by incorporating ammonia, a metabolic waste product, into the synthesis of glutamine, as done by several insect endosymbionts (Sabree *et al*., 2009; Hansen and Moran, 2011; Gerhart *et al*., 2018).

CMM genome encodes proteins that could assist *I. ricinus* in its response to oxidative stress and in energy metabolism (Olivieri *et al*., 2019) (Fig. 2). These proteins include the cytochrome cbb_3_ oxidases that may support ATP production with reduced levels of oxygen concentration during oogenesis, heme exporter proteins, a protoheme ferro-lyase, superoxide dismutase, ferrochelatase, nucleotide tlc translocases and a pathway for the synthesis of lipoic acid, a cofactor that forms part of diverse enzyme complexes of electron transport chains in mitochondria (Packer *et al*., 1995; Sassera *et al*., 2011; Olivieri *et al*., 2019). Proteins such as constituents of the major facilitator superfamily and the drug/metabolite transporter superfamily potentially responsible for the transportation of fluids and ions such as sodium, protons and potassium and the uptake of organic compounds like amino acids were also annotated in the genome of CMM suggesting that the bacterium may play a role in the maintenance of osmoregulation and water balance in *I. ricinus* during blood feeding (Olivieri *et al*., 2019). Lastly, a recently acquired CMM in *Hyalomma marginatum* seems to compensate for the loss of biotin and heme biosynthesis genes in the co-infecting FE (Buysse *et al*., 2021*b*), probably because both endosymbionts together provide critical metabolites to ticks as observed in several insects (Husnik and McCutcheon, 2016; Santos-Garcia *et al*., 2018; Takeshita *et al*., 2019).

## Experimental evidence for endosymbiont function

Administration of antibiotics that diminish endosymbiont populations leads to reduced reproductive success in ticks, suggesting a fitness-boosting role for endosymbionts (Fig. 2). For instance, exposure of *A. americanum* to tetracycline and rifampicin led to a decrease in CE load and concomitant reduction in reproductive fitness, as evidenced by a setback in the rate of oviposition and significantly lower number of ticks that emerged from eggs (Zhong *et al*., 2007). Similarly, injection of tetracycline into *R. microplus* and its cattle host led to reduced levels of CERM and a delay in the development of the tick at the metanymph phase (Guizzo *et al*., 2017), and administration of ofloxacin to *R. sanguineus* led to reduced burdens of CRs accompanied by an increase in time for adult females to feed to repletion, a reduced engorgement weight and a decreased capacity to lay eggs (Ben-Yosef *et al*., 2020; An *et al*., 2022). Likewise, tetracycline administration to female *H. longicornis* led to reduced densities of the *Coxiella* symbiont in the ovaries and malpighian tubules, which led to significant changes in tick engorgement weight, feeding time, number of eggs laid and the length of the oviposition period (Zhang *et al*., 2017).

The reduction in tick fitness associated with endosymbiont loss could be due to the diminished availability of B vitamins that are vital to tick development. Antibiotic treatment of *O. moubata* reduced the tick's FE burden and interrupted with normal nymph feeding and moulting, which forestalled the development of viable adult female ticks and significantly lowered the emergence of male ticks. Providing a B vitamin mixture (thiamine, riboflavin, niacin, pantothenic acid, pyridoxine, biotin, folate and cobalamin) to *O. moubata* restored its reproductive fitness, indicating a role for FLE-Om in provisioning B vitamins required for normal tick development and reproduction (Duron *et al*., 2018).

Experiments also suggest that endosymbionts may contribute to the blood-feeding capacity of ticks (Fig. 2). Treatment of *H. longicornis* nymphs with tetracycline reduced the levels of the *Coxiella* symbiont (CHI), which in turn reduced the blood intake of the tick (Zhong *et al*., 2021). Intriguingly, it is the metabolite chorismate produced by CHI rather than B vitamins or cofactors that likely influences tick blood intake. The study showed that chorismate promotes the production of 5-hydroxytryptamine (serotonin), a bioamine whose stability in the midgut and synganglion of the tick regulates the amount of blood ingested by the tick (Zhong *et al*., 2021). Similar to *H. longicornis*, administration of antibiotics to *R. sanguineus* and *R. microplus* also reduced the density of CEs and tick blood intake, and transcriptomic analysis of CERM-free *R. microplus* metanymphs revealed that genes associated with tick blood-feeding capacity such as DAP-36, lipocalin, trypsin inhibitor-like family, Kunitz-type inhibitors, cystatin and evasins were significantly under-expressed (Guizzo *et al*., 2017; Zhang *et al*., 2017; Ben-Yosef *et al*., 2020; An *et al*., 2022). Collectively, these data indicate that CEs improve blood feeding across tick species, but further studies are required to confirm whether endosymbiont-produced chorismate is the key metabolite that drives this process in all ticks.

CMM is the most common endosymbiont associated with *I. ricinus* and feeding females with tetracycline-containing bovine blood produced CMM-free ticks within 2 generations. Larvae that hatched from eggs laid by CMM-free females consistently performed poorly during blood feeding, suggesting that CMM is required for the emergence of larvae with intact blood-feeding ability (Guizzo *et al*., 2023). Similar to the above findings, *I. ricinus* nymphs fed with gentamicin-treated blood had significantly lower engorgement weights, lower moulting proportions and lower weights of moulted unfed adult females in comparison to nymphs fed on antibiotic-free blood (Militzer *et al*., 2023). These studies show that CMM, in addition to CEs, enhances blood intake by ticks. Interestingly, increased blood feeding by ticks seems to benefit the endosymbionts as well. For example, *Francisella* symbionts in *H. doenitzi* significantly increased in number during blood feeding, and *Rickettsia* sp. phylotype G021 and CMM multiplied massively in *I. pacificus* and *I. ricinus*, respectively, following blood meals (Sassera *et al*., 2008; Cheng *et al*., 2013; Liu *et al*., 2016). Thus, improved intake of blood, which nourishes both tick and endosymbiont, seems to be one of the major benefits of long-term symbiosis between ticks and intracellular bacteria.

Finally, *D. variabilis* and *A. americanum* larvae infected with *Rickettsia* symbionts displayed increased motility than uninfected larvae [42]. The locomotive ability of newly hatched larvae was determined on flat and inclined surfaces and *Rickettsia*-containing larvae displayed increased locomotive speed relative to uninfected larvae. Tick motility plays a role in host-questing success; thus, infection with *Rickettsia* symbionts may impact the disease risk posed by tick-borne pathogens.

## Conclusions and future directions

In summary, a major function of tick endosymbionts seems to be the provisioning of B vitamins, especially riboflavin, biotin and folate (Fig. 1). B vitamins are in short supply in vertebrate blood; hence, maintaining endosymbionts that reliably provide these vital nutrients could be an adaptation that allowed ticks to evolve a strictly blood-dependent lifestyle. Endosymbionts also seem to provide additional functions such as improved blood intake and increased motility that enhance tick physiology and reproductive success (Fig. 2). However, these roles have only been demonstrated in a few tick–endosymbiont systems, so more studies are needed to understand whether these features are lineage-specific or are more widespread. This information is especially relevant given that CE, FE and CMM phylogenies often do not agree with tick phylogenies (Epis *et al*., 2008; Cafiso *et al*., 2016; Duron *et al*., 2017; Binetruy *et al*., 2020). One of the underlying causes for this discordant evolutionary pattern could be that different tick species have acquired endosymbionts belonging to divergent *Coxiella*, *Francisella* and *Candidatus* Midichloria branches (Epis *et al*., 2008; Cafiso *et al*., 2016; Gerhart *et al*., 2018; Brenner *et al*., 2021). Thus, it is possible that depending on the lineages of their ancestors, endosymbionts in different tick species perform divergent functions.

Relatedly, another aspect of tick biology that we do not fully understand is how CEs and FEs evolved from pathogenic ancestors (Gerhart *et al*., 2016; Brenner *et al*., 2021). For example, pathogens such as *C. burnetii* and *F. tularensis* are not vertically transmitted (Genchi *et al*., 2015; Buysse *et al*., 2021*a*), but maternal transmission is a critical step in endosymbiosis. Thus, understanding how this process arose and is maintained in ticks would provide new insights into the biology of highly integrated tick–endosymbiont systems.

In addition to vertical transmission, an essential factor that sustains endosymbiosis is the presumed dependence of ticks on nutrients provided by endosymbionts. Going forward, functional studies to identify specific metabolites that sustain tick–endosymbiont relationships should be prioritized. Developing genetically tractable tick–endosymbiont model systems would accelerate this area of research by facilitating the disruption of endosymbiont genes to assess their impact on tick physiology and fitness. Although methodologies to culture and genetically manipulate CEs or FEs have not yet been developed, genetic tools and culture media are available for related pathogens *C. burnetii* and *F. tularensis* (Zogaj and Klose, 2010; Omsland *et al*., 2011). These systems could be adapted to generate mutant CE or FE strains to assay the contributions of specific metabolites to tick physiology and reproductive success.

Future functional studies should also device alternatives to the current practice of using antibiotics to generate endosymbiont-free ticks. This is because antibiotics may eliminate other members of the tick microbiota, thus making it difficult to determine whether any observed effect is solely due to the loss of the endosymbiont. Another key aspect to consider while investigating endosymbiont function is the potential contributions made by the rest of the tick microbiota towards tick physiology. For instance, gut microbiota may modify metabolites present in blood meal to make them amenable for use by tick or endosymbiont. Similarly, antibacterial peptides produced by the tick innate immune system in response to gut bacteria could impact the location and functions of tick endosymbionts (Narasimhan *et al*., 2021). Several recent studies have analysed the metabolic capabilities of tick microbiomes (Obregón *et al*., 2019; Estrada-Peña *et al*., 2020). These observations should be integrated with data from endosymbionts to gain a holistic view of how endosymbionts along with rest of the microbiota influence tick biology.

Lastly, targeting keystone taxa among tick microbiota is an innovative approach to inhibit the spread of ticks. Recent studies showed that microbiota were disrupted in ticks fed on blood from mice vaccinated against keystone taxa (Mateos-Hernández *et al*., 2020, 2021). While the exact physiological consequences of such ‘anti-microbiota’ vaccines are yet to be elucidated, this approach holds promise as an alternative to the use of acaricides to control tick infestation and pathogen transmission. A complementary approach would be to identify/develop molecules that target biochemical processes that are unique to endosymbionts, which are stable members of tick microbiota. For this approach to succeed, it is necessary to gain a deeper understanding of how the enmeshed tick–endosymbiont metabolic pathways function to improve tick physiology and fitness. By developing new molecular tools to manipulate endosymbiont genes and by considering endosymbiont physiology in the context of the whole tick microbiota, future studies should make anti-endosymbiont strategies a reality.

## Data Availability

Not applicable.
